# Neuroprotective Effect of Tea Polyphenols on Oxyhemoglobin Induced Subarachnoid Hemorrhage in Mice

**DOI:** 10.1155/2013/743938

**Published:** 2013-06-03

**Authors:** Haizhen Mo, Ying Chen, Liyong Huang, Hao Zhang, Juxiang Li, Wenke Zhou

**Affiliations:** ^1^Department of Food Science, Henan Institute of Science and Technology, Xinxiang, Henan 453003, China; ^2^School of Life Science and Technology, Henan Institute of Science and Technology, Xinxiang, Henan 453003, China; ^3^Department of Neurosurgery, The First Affiliated Hospital of Xinxiang Medical University, Weihui, Henan 453100, China

## Abstract

Tea polyphenols are of great benefit to the treatment of several neurodegenerative diseases. In order to explore the neuroprotective effects of tea polyphenols and their potential mechanisms, an established *in vivo* subarachnoid hemorrhage (SAH) model was used and alterations of mitochondrial function, ATP content, and cytochrome *c* (cyt *c*) in cerebral cortex were detected. This study showed that the alteration of mitochondrial membrane potential was an early event in SAH progression. The trend of ATP production was similar to that of mitochondrial membrane potential, indicating that the lower the mitochondrial membrane potential, lesser the ATP produced. Due to mitochondrial dysfunction, more cyt *c* was released in the SAH group. Interestingly, the preadministration of tea polyphenols significantly rescued the mitochondrial membrane potential to basal level, as well as the ATP content and the cyt *c* level in the brain cortex 12 h after SAH. After pretreatment with tea polyphenols, the neurological outcome was also improved. The results provide strong evidence that tea polyphenols enhance neuroprotective effects by inhibiting polarization of mitochondrial membrane potential, increasing ATP content, and blocking cyt *c* release.

## 1. Introduction

Polyphenols, the most abundant components in fruits, tea, wine, and vegetables, have attracted attention in recent years due to their healthy effects. It has long been known that tea polyphenols have neuroprotective effects in various pathological states of the nervous system, for example, by lowering the cognitive impairment in most neurodegenerative diseases and by reducing the risk of mortality after stroke [1–3]. Human epidemiological data shows that the risk of a fatal versus nonfatal stroke is significantly reduced by approximately 21% in people that drink 3 cups of tea per day when compared with nontea drinkers. In fact, high consumption of tea, particularly green tea, contributes to the lower rates of cardiovascular disease observed in the Asian population, especially in China, indicating that tea drinking may be a benefiting lifestyle. However, other authors indicate that tea consumption has no correlation with hemorrhagic stroke [4]. 

Stroke is a leading cause of morbidity and mortality worldwide. Subarachnoid hemorrhage (SAH) accounts for only 5%–10% of all strokes but is a major devastating subtype of stroke affecting 30,000 people in North America yearly [5]. SAH is caused by the rupture of a brain aneurysm and can be divided in two stages, the first 72 h correspond to the early brain injury (EBI) and the period after 72 h corresponds to the delayed vasospasm. About 21% of SAH survivors do not experience delayed vasospasm indicating that EBI may be an important stage predicting the outcome of SAH [6, 7]. In fact, all the factors related to the pathological mechanisms of EBI after SAH may eventually induce irreversible neuronal death, which is associated with neurological deficits and poor outcome [8–10]. 

A growing body of evidence from animal models and clinical studies indicate that mitochondrial dysfunction is a common event in brain injury, including in SAH [11]. Several lines of research suggest that mitochondrial dysfunction, induced by oxidative stress and inflammation, results in mitochondrial membrane potential (Δ*ψ*_*m*_) reduction, cytochrome *c* (cyt *c*) release, and then activation of mitochondrial-dependent cell death, providing evidence that mitochondrial impairment is deleterious [12, 13]. Therefore, mitochondrial function is a potential therapeutic target [14].

Recent results suggest that inhibition of a signal pathway may not be required for the treatment of SAH [15]. In such a scenario, tea polyphenols may be of special interest due to their multiple functions, such as antioxidants, antimutagenic, iron chelators, and in glutamate release [16]. However, tea polyphenols' effects in the time course after SAH are still unknown. The aim of this study was to investigate whether tea polyphenols have neurological and neurobehavioural effects or not, and the pathway involved in these effects in the *in vivo* SAH model. This study contributes to a better understanding of the role of tea polyphenols in SAH prevention, which are nontoxic and inexpensive dietary components.

## 2. Materials and Methods

### 2.1. Preparation Oxyhemoglobin (OxyHb)

Arterial blood collected from Kunming mouse with heparin was centrifuged at 2,500 ×g for 15 min. The supernatant was discarded. Erythrocytes were washed 3 times with saline solution, lysed with methylbenzene, and centrifuged at 15,000 ×g for 20 min; the supernatant was collected. Using filter membrane, the OxyHb solution was collected, adjusted to 3 *μ*mol/L using measured optical density (OD) at 540 nm and 576 nm, and stored at −80°C [17].

### 2.2. Animals

The animal use and care protocols were approved by Institutional Animal Care and Use Committee (IACUC) of Xinxiang Medical University. One hundred and eighty adult male Kunming mice weighing from 18 to 20 g were purchased from Xinxiang Medical University. All animals were required to undergo institutional quarantine for 7 days prior to use. The environment for animal housing was equipped with controlled temperature (22 ± 3°C), humidity (40%–70%), and a 12 h light/dark alternation. The mice were divided into three groups: sham group (*n* = 60), SAH group (*n* = 60), and preadministration tea polyphenols SAH group (*n* = 60). Subsequently, each group was subdivided into 3 h, 6 h, 12 h, 24 h, and 72 h subgroup (*n* = 12), respectively. SAH group was injected with OxyHb, and sham group was given isotonic saline for the same period. Pretreatment group was administered intragastrically with water containing tea polyphenols at a dose of 450 mg/kg/d for 7 days before OxyHb injection [18, 19]. 

### 2.3. Mouse SAH Model

SAH was performed using the model reported by Shi et al. [20]. Under general anesthesia with 1% pentobarbital (50 mg/kg, intraperitoneally), animals were placed in prone position. The posterior cervical muscles were dissected through a suboccipital midline skin incision and retracted laterally. The exposed transparent atlantooccipital membrane was penetrated by a 30 gauge needle. Under spontaneous breathing, a 23-gauge needle without point was inserted percutaneously into the skull at a controlling depth of 1.5 mm in the cross position at the sagittal suture 2 mm and sutura coronaria 1 mm. Then, 50 *μ*L (150 *μ*mol/L) of OxyHb was injected through this hole into subarachnoid space. After the neurological assessment, the animals were decapitated at different time points after SAH. Prior to decapitation, one part (*n* = 6) was perfused through the left cardiac ventricle with isotonic saline, followed by 4% paraformaldehyde in phosphate-buffered saline (PBS). The brain tissue was removed and fixed in 4% paraformaldehyde for 48 h and then embedded in paraffin. Sections of 4 mm thickness were cut using a microtome for histologic studies. The others (*n* = 6) were sacrificed with 0.9% saline solution perfusion through the left cardiac ventricle. The fresh brain was immediately removed and cortex sections selected and then stored at −80°C. 

### 2.4. Mortality and Neurological Functions Assessment

Recently, the Garcia scoring system has been developed to evaluate the animal neurological behavior and function in a blinded fashion [21]. The results showed that the higher the neurological score, the better the outcome. Briefly, the neurobehavioral examination was performed at 6 h, 12 h, 24 h, and 72 h. An 18-point scoring system was used based on (1) spontaneous activity, (2) symmetry of limb movement, (3) climbing, (4) body proprioception, (5) movement of forelimbs, and (6) response to vibrissae touch (score scale: 0–3 each).

### 2.5. Lactate Dehydrogenase (LDH) Assay from Brain Cortex

The supernatant of all the samples was collected after homogenate and the LDH content was determined using an LDH assay kit according to the manufacturer's instructions (Nanjing Institute of Jiancheng Biological Engineering, China) [22]. LDH cytotoxicity was calculated using OD as LDH cytotoxicity (U/g protein) = (OD sample − OD blank)/(OD standard solution − OD blank standard solution) × standard solution concentration/sample protein concentration.

### 2.6. Isolation of Mitochondria from Cerebral Cortex

Intact mitochondria were isolated from fresh brain cortex layer using a tissue mitochondria isolation kit (Beyotime Institute of Biotechnology, China). In brief, after homogenization of 0.5 g cortical tissue in ice-cold MSH buffer (10 mM HEPES, pH 7.5, containing 200 mM mannitol, 70 mM sucrose, 1.0 mM EGTA, and 2.0 mg/mL serum albumin), the homogenate was centrifuged at 1,000 ×g at 4°C for 10 min. The collected supernatant was then centrifuged at 3,500 ×g at 4°C for 10 min to obtain a mitochondrial pellet [23]. 

### 2.7. Assay of Mitochondrial Membrane Potential

Changes in mitochondrial membrane potential (Δ*ψ*_*m*_) were measured using a JC-1 (5′,6,6′-tetrachloro-1,1′,3,3′-tetraethylbenzimidazolylcarbocyanide iodide) staining (mitochondrial membrane potential assay kit, Beyotime Institute of Biotechnology, China) according to the manufacturer's instructions. Briefly, isolated mitochondria were suspended in 0.5 mL medium containing 5 mM JC-1. Samples were analyzed using an automatic microplate reader (Thermo Scientific, USA) at time scan method. The intensities of green (excitation/emission wavelength = 485/538 nm) and red (excitation/emission wavelength = 485/590 nm) fluorescence were analyzed in each sample and represented a surrogate marker of loss of mitochondrial Δ*ψ*_*m*_ [24]. 

### 2.8. Assay for Cellular ATP Levels from Brain Cortex

After being thawed, samples were homogenized in boiling double distilled water in order to denature endogenous ATPase present in the tissue. The supernatant fraction of the homogenate was collected after centrifugation at 10,000 ×g for 10 min. ATP levels were measured using ATP colorimetric assay kit according to manufacturer's instructions (Nanjing Institute of Jiancheng Biological Engineering, China) [25]. 

### 2.9. Immunohistochemistry for Cyt *c* Antigen

Deparaffinized sections were treated with 0.3% hydrogen peroxide in methanol for 15 min at room temperature to block endogenous peroxidase activity. The sections were incubated in 0.01 M, pH 6.5 sodium citrate buffer for 10 min at 121°C, and cooled to room temperature. After being blocked with 10% normal goat serum for 1 h at room temperature, the slides were subsequently incubated overnight with anti-cyt *c* antibody (BA0781, Boster Bio-Engineering Limited Company, China) at a dilution of 1 : 100. After being extensively washed with PBS, the slides were incubated with Histostain-Plus kit (SP-9001, Zymed, USA). The sections were then counterstained with DAB (ZLI-9032, Zhongshan Golden Bridge Biotechnology Co., LTD, China). Quantitative evaluation was measured using IDA-2000 software (Beijing Konghai Technology Company, China). At least 10 visual fields were captured and more than 500 cells were counted [26, 27]. 

### 2.10. Statistical Analysis

The statistical analysis was performed using the Statistical Package for the Social Sciences (SPSS Inc., Chicago, IL, USA) program. All data were reported as means ± SD of three independent experiments. The physiological variables were analyzed by one-way ANOVA followed by LSD multiple comparison post hoc analysis. The neurological scores were compared by Kruskal-Wallis nonparametric test followed by multiple comparison procedures by Duncan's method. For all comparisons, *P* < 0.05 was considered statistically significant.

## 3. Results

### 3.1. Mortality and Neurological Scores

Of a total of 180 mice, 8 (4.4%) died over the course of the experiment, 5 (8.3%) in the SAH group, 3 (5.0%) in the tea polyphenols pretreated animals, and 0 in the sham group. This suggests that tea polyphenols pretreatment reduced the mortality in consequence of SAH.

**Figure 1:**
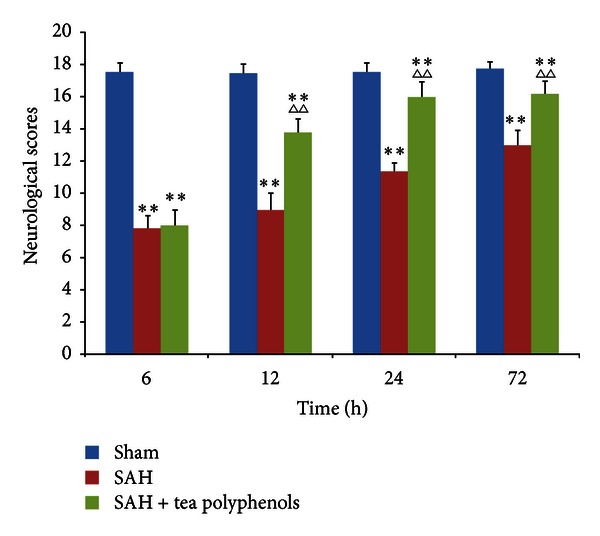
Neurological deficits in OxyHb-induced SAH mice. Values are expressed as mean ± SD of triplicate samples. **P < 0.01 versus sham; ^∆∆^ *P* < 0.01 versus SAH.

The neurological scores obtained for sham, SAH, and tea polyphenols + SAH groups are depicted in Figure 1. The neurological score observed for mice with SAH was significantly lower than that of the sham group from 6 h to 72 h after SAH (*P* < 0.01 versus sham), whereas the improved neurological scores were observed after 12 h of SAH in animals pretreated with tea polyphenols pretreatment group (*P* < 0.01 versus SAH), indicating that tea polyphenols rescued neuronal injury. 

### 3.2. The Neuroprotective Role of Tea Polyphenols in Early Brain Injury after SAH

**Figure 2:**
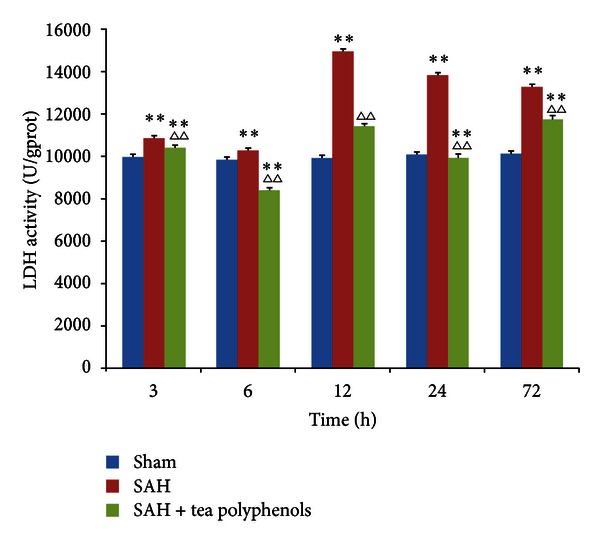
Effect of tea polyphenols on LDH activity in OxyHb-induced SAH. Data are expressed as the mean ± SD of three independent experiments. **P < 0.01 versus sham; ^∆∆^ *P* < 0.01 versus SAH.

LDH activity is the most widely used marker in cytotoxic studies. Using this assay, we detected a neuroprotective role for tea polyphenols in the EBI stage after SAH. LDH activity was stable in the cortical tissue of the sham group (Figure 2). After SAH, LDH levels were significantly increased in the cortex along with the SAH progress (*P* < 0.01 versus sham), but the peak level was observed 12 h after SAH, indicating that LDH activity might be time dependent. However, pretreatment with tea polyphenols led to a significant decrease in LDH activity compared to the SAH group (*P* < 0.01 versus SAH). A significant difference was observed between groups pretreated with tea polyphenols and sham (*P* < 0.01 versus sham), except at 12 h. LDH levels after pretreatment with tea polyphenols were variable compared to those detected in the sham group.

### 3.3. The Preventive Effect of Tea Polyphenols on Mitochondrial Depolarization in the Cortex after SAH

**Figure 3:**
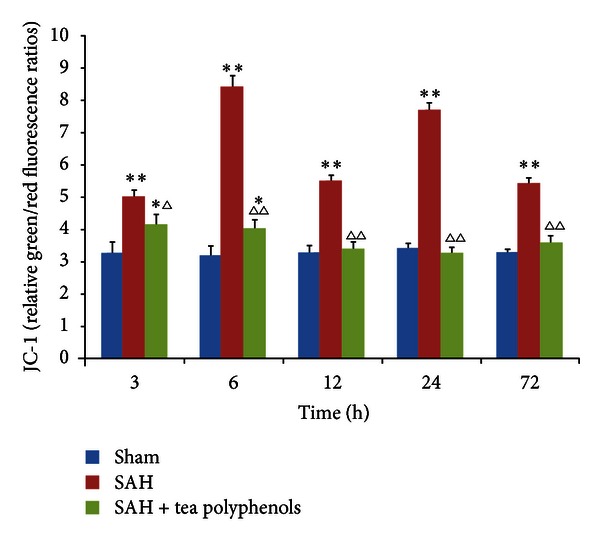
Effect of tea polyphenols on the mitochondrial membrane potential. Data are expressed as the mean ± SD of three independent experiments. *P < 0.05 versus sham; **P < 0.01 versus sham; ^∆^ *P* < 0.05 versus SAH; ^∆∆^ *P* < 0.01 versus SAH.

Mitochondrial membrane potential (Δ*ψ*_*m*_), a widely recognized biomarker of mitochondrial function, can be measured using a cationic lipophilic dye, JC-1. OxyHb caused significant mitochondrial membrane depolarization in the SAH group, which was expressed as an increase in JC-1 green/red fluorescence ratios (*P* < 0.01 versus sham) (Figure 3). However, the preadministration of tea polyphenols prevented the loss of mitochondrial membrane potential (*P* < 0.05 versus sham); 12 h after SAH, no significant difference was observed compared with the sham group (*P* > 0.05 versus sham). The decrease of mitochondrial membrane potential in SAH group was greatly alleviated by tea polyphenols pretreatment with a time-dependent effect (*P* < 0.05 versus SAH).

### 3.4. Tea Polyphenols Increasing ATP Content in the Development of SAH

**Figure 4:**
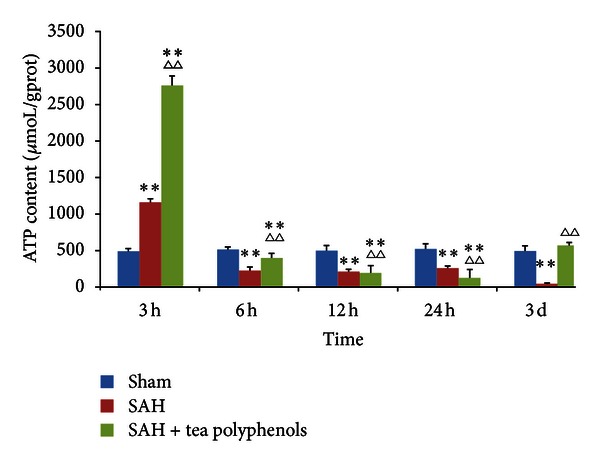
Alteration of ATP content after SAH. All the experiments were performed as described in Section 2. Data are expressed as the mean ± SD of three independent experiments. **P < 0.01 versus sham; ^∆∆^ *P* < 0.01 versus SAH.

After SAH, ATP content was determined using a validated ATP detection assay in the cortex (Figure 4). Within the cortical region, the steady-state ATP level was stable in the sham group at different time points. However, ATP levels were dramatically altered both in the SAH and the tea polyphenols pretreatment groups with a distinct pattern. Interestingly, the ATP content increased rapidly, showing the highest values 3 h after SAH both in the SAH and the tea polyphenols pretreatment groups (*P* < 0.01 versus sham). After 3 h of SAH, ATP levels dramatically declined and the lowest levels were observed at 3 days after SAH, suggesting a time-dependent ATP depletion (*P* < 0.01 versus sham). In the tea polyphenols treatment group, a fluctuation in the ATP level was observed. ATP levels were gradually reduced from 6 h to 24 h, whereas a significant increase in ATP content was observed 3 days after SAH (*P* > 0.05 versus sham, *P* < 0.01 versus SAH). In conclusion, tea polyphenols significantly increased ATP content after SAH.

### 3.5. Effect of Tea Polyphenols on the Cyt *c* in EBI after SAH

**Figure 5:**
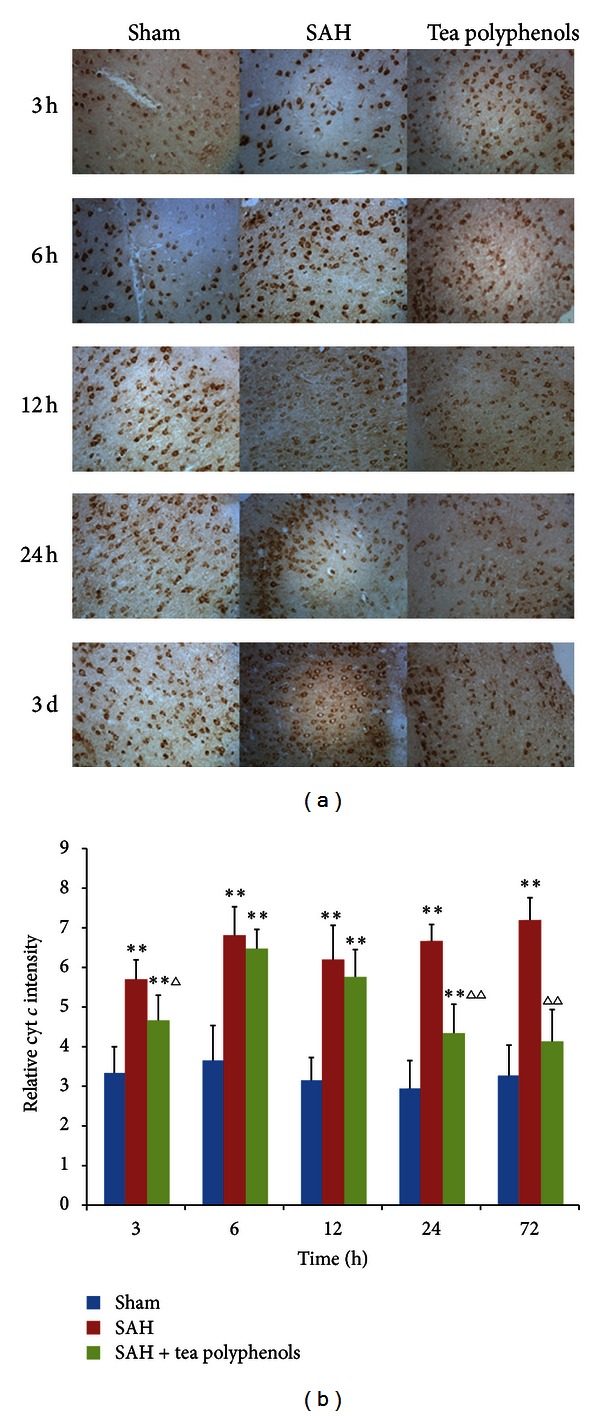
Tea polyphenols prevented cyt *c* release. (a) Identification of cyt *c* by immunohistochemical assay (×400). (b) Quantification of cyt *c *level by densitometry. At least 10 visual fields were captured and more than 500 cells were counted. The quantification represents means and standard deviations of results from three independent experiments. **P < 0.01 versus sham; ^∆^ *P* < 0.05 versus SAH; ^∆∆^ *P* < 0.01 versus SAH.

Considering that cytoplastic cyt *c* level is increased along with depolarization of mitochondrial membrane potential, cyt *c* content was measured in the different experimental models using immunohistology. As shown in Figure 5(a), a moderate and stable immunoreactivity was observed in the sham group, whereas robust cyt *c* levels were observed at all time points after SAH. However, after pre-administration of tea polyphenols, cyt *c* levels were gradually decreased to basal level. The cyt *c* levels were significantly increased in the SAH group compared to the sham group (*P* < 0.01 versus sham) (Figure 5(b)). Interestingly, cyt *c* values after pre-administration of tea polyphenols were higher than that of sham, but lower than that of SAH group. Significant difference was observed from 3 h to 24 h of sham (*P* < 0.01 versus sham), as well as 3 h, 24 h and 3 days of SAH (*P* < 0.05 versus SAH), indicating that tea polyphenols can block cyt *c* release after SAH.

## 4. Discussion

Mitochondria contribute to many cellular events involving intracellular calcium homeostasis, reduction-oxidation potential, cell cycle regulation, and synaptic plasticity [28]. There is a current awareness that mitochondria are highly likely subjected to insults; therefore, mitochondrial dysfunction may act as one of the main trigger events in central nervous system disease, such as Parkinson's disease, Alzheimer's disease, and ischemic stroke, leading to cell death, and ultimately diseased brain [29–31]. The depolarization of the mitochondrial membrane potential (Δ*ψ*_*m*_) is a common event in mitochondrial dysfunction through which apoptosis, necrosis, and autophagy can be driven [32, 33]. Our results show a significant decrease in Δ*ψ*_*m*_ in the SAH group compared with sham group. Several studies have shown that tea polyphenols significantly prevent cell swelling and the decline in the Δ*ψ*_*m*_ [34]. In line with these reports, we observed that in the pretreated tea polyphenols group, the Δ*ψ*_*m*_ gradually increased to basal level 12 h after SAH indicating that one mechanism by which tea polyphenols exert their protective effects is possibly by inhibition of the depolarization of the inner membrane potential. Due to lack of glycolytic capacity, more mitochondria are required to produce the necessary energy in the brain than that in other organs [35]. In pathological conditions, loss of Δ*ψ*_*m*_ leads to the mitochondrial permeability transition (MPT) pore opening and to osmotic swelling of the mitochondrial matrix and to defective oxidative phosphorylation, thus impairing ATP synthesis. After SAH, the ATP levels were decreased compared to sham controls but could be restored by tea polyphenols pretreatment, indicating that tea polyphenols may block mitochondrial dysfunction followed by increased ATP content, eventually leading to neuronal cell survival.

Since the brain is highly sensitive to changes in mitochondrial respiration due to its higher consumption of oxygen and fewer free radicals scavenger ability, changes in Δ*ψ*_*m*_ may also ultimately lead to apoptosis through downregulation of antiapoptotic proteins, as well as activation of proapoptotic pathways [36]. More specifically, mitochondrial dysfunction would inhibit cytochrome *c* oxidase activity and lead to release of cyt *c* into the cytosol, which initiates the caspase cascade. There is no doubt that mitochondrial respiratory dysfunctions leading to low Δ*ψ*_*m*_ might result in the release of pro-apoptotic proteins, such as the apoptosis inducing factor (AIF) and cyt *c *[37]. The latter activates caspase-9, thus activating caspase-3-dependent apoptotic pathway. In addition, in low Δ*ψ*_*m*_ conditions, PINK1 accumulates on the surface of the mitochondria and recruits Parkin, an ubiquitin ligase, which ubiquitinylates Bcl-2, further inducing cyt *c* related and apoptosis [38, 39]. It has been shown that increased cyt *c* mediates DNA fragmentation and apoptosis in mouse brains in subarachnoid hemolysate [40]. In other words, with SAH progression, neuronal cells enter the apoptotic pathway in the cortex. However, tea polyphenols can significantly inhibit cyt *c* release by blocking mitochondrial dysfunction at the EBI stage after SAH. This finding is supported by previous results that showed that the number of apoptotic cells was reduced after using green tea extract for pre-treatment of ischemia in gerbils [41].

## 5. Conclusion

In the present study, we found that neuroprotective effects of tea polyphenols may rely on their mitochondrial protection behavior. Our studies showed that tea polyphenols pretreatment reduces mitochondrial dysfunction markers and increases neurological scores after SAH [42, 43]. This suggests that dietary supplementation with tea polyphenols could be a potential candidate for prevention of SAH. As tea polyphenols represent a class of natural, dietary components, further research will be necessary to better identify which polyphenols play the roles in SAH, and which signaling pathways are involved in these neuroprotective effects.
