# EXPRESSION OF CONCERN: Neuroprotective Effect of Tea Polyphenols on Oxyhemoglobin Induced Subarachnoid Hemorrhage in Mice

**DOI:** 10.1155/omcl/9848436

**Published:** 2026-03-23

**Authors:** Oxidative Medicine and Cellular Longevity

**EXPRESSION OF CONCERN**: H. Mo, Y. Chen, L. Huang, H. Zhang, J. Li, and W. Zhou, “Neuroprotective Effect of Tea Polyphenols on Oxyhemoglobin Induced Subarachnoid Hemorrhage in Mice,” *Oxidative Medicine and Cellular Longevity* 2013, 743938, https://doi.org/10.1155/2013/743938.

This Expression of Concern is for the above article, published online on 03 June 2013 in Wiley Online Library (wileyonlinelibrary.com), and has been issued by agreement between the journal’s Editor‐in‐Chief Jeannette Vasquez‐Vivar; and John Wiley & Sons Ltd. A third party reported on PubPeer [1] that the 3H SAH image overlapped with the 6 h Sham image in Figure 5. This concern was confirmed by the publisher.

The authors did not respond to an inquiry by the publisher and request for original data.

The Expression of Concern has been agreed to in order to alert readers to the concerns in Figure 5. The authors have been informed of the Expression of Concern.
